# Supplementary material for: Efficacy of mefloquine and its enantiomers in a murine model of Mycobacterium avium infection
Source: PLoS One. 2024 Sep 30;19(9):e0311167. doi: 10.1371/journal.pone.0311167 (PMC11441642; doi:10.1371/journal.pone.0311167)
Supplement: Attachment — Submitted filename: Response to reviewers.docx [file pone.0311167.s002.docx]

Reviewer #1: Froment and colleagues determined the efficacy of mefloquine and its enantiomers using a murine model of pulmonary M.avium infection.

In the reported study, the investigators infected mice using an aerosol device for bacterial delivery and after 28 days treatment was initiated. The mice were treated with rifampin, clarithromycin, and ethambutol or clarithromycin, ethambutol and mefloquine (racemic mixture) or substituting the mefloquine in the regimen for + or – mefloquine enantiometer, daily, for 3 months.

The results suggest that the combination containing mefloquine (+) was the most active, although the results did not reach statistically significance.

Comments and Suggestions:

1. In the experimental design the group of animals that did not received treatment, was not described (methods section). Did they received similar amount of ethanol that was used to dissolve the compounds ? The description of methods can be improved.

Thank you for this comment. As ethanol is known to augment M. avium virulence and decrease macrophages function toward mycobacteria (Bermudez, Alcohol 1994; Bermudez, JID 1993) we did not expose untreated mice to ethanol. Furthermore, to ease the burden on mice and on experimenters we did not performed gavage on untreated mice. We added some precisions in the method section (line 126).

2. The results obtained suggest that all treatments groups were equally effective, which is not a surprise. The difference in CFUs is not significant, at months 1, 2 and 3 in the same treatment group and at a longitudinal comparison between groups. Did the authors tested for antibiotic resistance among the survival strains?

After combination therapy, we did not expect to find resistance acquisition after 1 to 3 months of treatment. Therefore, we did not test for resistance. However, it would be of interest to add treatment groups with 2—drug combination and longer treatment duration and test for resistance acquisition.

3. The number of animals used per time pint is quite small. I a couple of experimental groups one of the mouse was lost (the authors made no mention about the reason), which make the analysis difficult, in despite of the large difference in the number of CFU comparing treated and untreated mice.

Thank you for this comment. The number of animal per group was calculated as follow: α risk =5%, 1-β risk =80%, standard deviation 0.25 (from past studies). Therefore, groups of 4 mice would be enough to show a significant difference of 0.5log10 between groups. To account for unfortunate loss of animal we used 5 mice per group. Unfortunately, 2 mice died during the first month lowering statistical power. However, this affected the first month only. We also saw an inter-individual difference (standard deviation) more important than expected, lowering our statical power.

4. The discussion of the manuscript needs to touch in many other points, regarding the disease and treatment. Mefloquine is used for the treatment of malaria. How it fits with the anti-bacterial action?

Thank you for this comment; we improved the discussion in this direction. Mefloquine is known to have anti-mycobacterial effect (Bermudez, JID 2008). The mechanism of action seems to be an inhibiting activity on DNA-synthase and a mycolic acid transporter (Upadhayaya, Eur J Med Chem 2010, Degiacomi Int J Mol Sci 2021).

5. The legend of figure 1 needs to be improved, with better description of the experiments and definition of symbols.

Thank you for this remark; we clarified the legend in the revised manuscript.

Reviewer #2: The authors show, very succinctly, interesting results of an animal model of Mycobacterium avium infection and various 3 drug cocktails, including Mefloquine. Curiously, the cfu counts rebound over time, with the caveat that they had issues with the third month cultures. The authors show that they can do the drug treatments and successfully used an aerosol model of infection.

However, this very brief collection of preliminary results is essentially a promising pilot study. It includes a table that simply recapitulates the data shown in the graphs. Further, while this pilot study shows promise, the authors themselves admit to an issue with culturing the bacteria on the 3rd month. While not a disqualifying issue, it further emphasizes the need to replicate these result, including using larger groups of mice. We also would need more information on the pathology of lung, data on the cell subsets found, and, likely, followup for long term infections to see if the bacteria do become completely drug resistant. Adverse effects in the mice are not shown. There are numerous issue with grammar that need correction as well.

Thank you for this comment. Although, we agree that this study is not sufficient in itself to demonstrate the interest of the mefloquine in MAC infections, we believe that these results, while waiting to be replicated, can motivate other researchers to pursue this treatment option.

Reviewer #3: General comments

MFQ has been considered for the treatment of infections by M. avium and other mycobacteria. Studies by Bermudez et al. previously demonstrated that MFQ and its enantiomers (administered by gavage) have efficacy in mouse models of M. avium infection (refs 14 and 15 in the manuscript submitted by Froment et al. and PMID 12792877, which is not cited in the manuscript). Some of the work reported by Bermudez et al. included drug combinations, e.g., MFQ, EMB, and moxifloxacin in PMID: 12792877 or MFQ and EMB in ref 14. In these experiments, mice were infected intravenously (caudal vein), and bacterial loads (CFU) were quantitated in blood, liver, and spleen. However, bacterial loads in lung or lung gross pathology were not evaluated.

In the study submitted by Froment et al., the authors extend the investigation of MFQ and its enantiomers ((+)-EMQ and (-)-EMQ) efficacy using a model of M. avium pulmonary infection. They compared the efficacy of MFQ as a replacement for RIF in the CLR-EMB-RIF gold standard regimen. Their use of an aerosol model of M. avium pulmonary infection for treatment efficacy assessment is a significant contribution and contrasts with the previously reported models noted above. The replacement of RIF with MFQ in the CLR-EMB-RIF gold standard regimen is also novel. Based on their results, the authors conclude that MFQ and its enantiomers appear to be as effective as RIF in combination therapy.

The urgency for new and alternative treatments for M. avium infections cannot be overstated, making the subject of this study not only important but also highly relevant for PLOS ONE.

Major points

The conclusion is based on a single and relatively short experiment with three data points for bacterial loads in the lungs, one of which could have been compromised (month 3). Additional experiments are required to increase the robustness of the conclusion and to expand the significance of the contribution made by this manuscript. The authors should repeat the study. Extension of the study beyond three months and analysis of lung gross pathology should be added to the study.

Thank you for this comment. Although, we agree that this study is not sufficient in itself to demonstrate the interest of the mefloquine in MAC infections, we believe that these results, while waiting to be replicated, can motivate other researchers to pursue this treatment option.

What the bacterial loads in the lung would have been without RIF in the authors' experimental model is unknown. Is there a statistically significant difference between RIF-CLR-EMB and CLR-EMB in the authors' experimental model? If the answer is no, then MFQ and its enantiomers might have no impact on lung CFUs. A CLR-EMB treatment group should be included in the study to address this uncertainty.

Thank you. This is a very interesting point. We will include a CLR-EMB control group in our replicate study.

A question the authors could also address to expand their study's contribution is whether MFQ or its enantiomers alone impact lung CFUs. Is the pharmacokinetic of MFQ and its enantiomers in the mouse model used known? What concentration of MFQ and its enantiomers do the authors expect in lung tissue, and how do those concentrations relate to the MICs against M. avium?

There is no PK data for lung in the literature in the case of MAC pulmonary infections. However, in a study of PK in an uninfected mouse model (Chung et al, Pharmacology 1982) the pulmonary distribution of Mefloquine is high and always higher than the MIC of 16µg/mL found in the study by Bermudez, Antimicrob. Agents Chemother 1999. So, theoretically the lung concentrations are 4 times the MIC.

Minor points

Does Table 1 show differences +/- SD or SEM? How were SD/SEM calculated?

It represents the standard deviation of the mean difference calculated by the formula

How was enantiomeric purity assessed?

We assessed this purity by a column chromatography technique. We made the precision in the manuscript (line 98)
