# Supplementary material for: Efficacy of mefloquine and its enantiomers in a murine model of Mycobacterium avium infection
Source: PLoS One. 2024 Sep 30;19(9):e0311167. doi: 10.1371/journal.pone.0311167 (PMC11441642; doi:10.1371/journal.pone.0311167)
Supplement: Attachment — Submitted filename: Response to reviewers.docx [file pone.0311167.s003.docx]

Reviewer #2: This revised version of this Short Communication appears to have generally responded as much as needed to the prior critiques. There is precedent in this journal for the publication of similar brief studies, thus it doesn't appear necessary to the editors to ask for some of the major revisions asked for by the previous reviews.

We thank you for this review, we have shortened our manuscript to fit the requirements of a brief communication

Reviewer #3: The authors have not adequately addressed major points of concern. These concerns remain in the revised manuscript. The work still requires major revisions noted in the previous comments by this reviewer. The presented work is still viewed as a pilot study presenting interesting preliminary results that warrant adequate experimental follow-up and expansion to consolidate conclusions and provide a robust contribution to the field. I would encourage the authors to continue and expand this work to address major points of concern and resubmit their work for consideration.

Thank you for this review, further work is planned but more funding is required.
